# Expression of G protein-coupled receptors and related proteins in HEK293, AtT20, BV2, and N18 cell lines as revealed by microarray analysis

**DOI:** 10.1186/1471-2164-12-14

**Published:** 2011-01-07

**Authors:** Brady K Atwood, Jacqueline Lopez, James Wager-Miller, Ken Mackie, Alex Straiker

**Affiliations:** 1 Department of Psychological & Brain Sciences, The Gill Center for Biomolecular Science, Indiana University, Bloomington, Indiana, USA; 2 Center for Genomics and Bioinformatics, Indiana University, Bloomington, Indiana, USA; 3 Graduate Program in Neurobiology and Behavior, University of Washington, Seattle, Washington, USA

## Abstract

**Background:**

G protein coupled receptors (GPCRs) are one of the most widely studied gene superfamilies. Thousands of GPCR research studies have utilized heterologous expression systems such as human embryonic kidney cells (HEK293). Though often treated as 'blank slates', these cell lines nevertheless endogenously express GPCRs and related signaling proteins. The outcome of a given GPCR study can be profoundly influenced by this largely unknown complement of receptors and/or signaling proteins. Little easily accessible information exists that describes the expression profiles of the GPCRs in cell lines. What is accessible is often limited in scope - of the hundreds of GPCRs and related proteins, one is unlikely to find information on expression of more than a dozen proteins in a given cell line. Microarray technology has allowed rapid analysis of mRNA levels of thousands of candidate genes, but though often publicly available, the results can be difficult to efficiently access or even to interpret.

**Results:**

To bridge this gap, we have used microarrays to measure the mRNA levels of a comprehensive profile of non-chemosensory GPCRs and over a hundred GPCR signaling related gene products in four cell lines frequently used for GPCR research: HEK293, AtT20, BV2, and N18.

**Conclusions:**

This study provides researchers an easily accessible mRNA profile of the endogenous signaling repertoire that these four cell lines possess. This will assist in choosing the most appropriate cell line for studying GPCRs and related signaling proteins. It also provides a better understanding of the potential interactions between GPCRs and those signaling proteins.

## Background

The G protein-coupled receptor (GPCR) gene superfamily consists of hundreds of members that are widely expressed in all tissues and serve as receptors for a diverse complement of ligands. Approximately 100 of these receptors are considered orphan GPCRs, in that no endogenous ligand has been confirmed for them [1]. Characterized by having seven transmembrane alpha-helical domains [2], GPCRs mediate a wide spectrum of cellular processes ranging from cell growth to neurotransmission [1-3]. A number of classification systems for GPCRs have been developed as bioinformatic tools[3-5]. One of the most commonly used GPCR classification systems, the one set forth by Kolakowski and developed by Vriend [4,6,7], groups GPCRs into six classes based on structural similarities to prototypical receptors. Class A GPCRs are the most abundant (>80% of all GPCRs), defined by a high sequence homology to rhodopsin, and include the chemosensory receptors. Class B GPCRs are also known as secretin-like receptors. Class C GPCRs are the metabotropic glutamate-like receptors. Class F/S receptors consist of frizzled/smoothened receptors, though there is debate about their ability to couple to G proteins [2]. Class D/Fungal pheromone and Class E/cAMP receptors are absent in mammals.

On activation via ligand binding, GPCRs couple to heterotrimeric G proteins composed of three subunits: Gα, β and γ. G protein α subunits are classified as members of one of four groups: G_i/o_, G_s_, G_q_, and G_12/13 _[8]. Each subunit couples to specific cellular protein targets (e.g. adenylyl cyclases) through which cellular activity is modulated. The Gβ/γ G protein subunits are capable of acting at their own specific targets, such as modulating ion channel activity [9-11]. The available complement of heterotrimeric G proteins will therefore affect its response profile.

A regulatory system controls the timing and duration of GPCR signaling. GPCR kinases (GRKs) phosphorylate agonist-occupied receptors, typically leading to receptor desensitization. Beta-arrestins are scaffolding proteins that are recruited to activated GPCRs and prevent G protein association with the receptor. They also serve to spatially segregate GPCR signaling to molecules such as mitogen-activated protein kinases [12,13]. The Regulators of G protein signaling (RGS) proteins, which enhance the GTPase activity of Gα subunits, is another large family of proteins important in fine-tuning GPCR signaling [14,15].

Due to their central roles in many cellular functions, GPCRs are important therapeutic targets. Different reports estimate that between 30 and 50% of all prescription drugs target GPCRs. Interestingly, these drugs only target ~10% of the non-chemosensory receptors, leaving hundreds of GPCRs as potential drug targets [2,9,16].

Identifying ligands, studying receptor protein interactions, and measuring cellular effects of receptor activation is often achieved by heterologous expression of GPCRs in cell lines such as HEK293 and AtT20 [17,18]. They also serve as models of cell types from which they are drawn (e.g. BV2 for microglia and N18 for neurons) [19-21]. However these cell lines have remained something of a black box; researchers make use of them without full knowledge of the complement of GPCRs and related proteins. The presence of an unknown protein in these cell lines may confound an otherwise sound series of experiments and differential expression of GPCRs and their regulatory proteins may explain differences in reported results across cell lines [13,17,22-25]. It is also clear that GPCRs can form oligomers with other GPCRs. These oligomers may confer different signaling properties to a transfected GPCR of interest, and can add layers of variability and complexity.

Efforts to define expression of GPCRs, on a receptor-by-receptor basis, using polymerase chain reaction (PCR)-based methods have only scratched the surface. Even for the widely used HEK293 cells, the expression of fewer than thirty GPCRs has been described [17,26]. Recently developed high-throughput methods have made possible the simultaneous assay of mRNA levels for thousands of genes. Microarray analyses have matured sufficiently in terms of accessibility and cost to make their application to this question tractable. We are not the first to make use of microarray technology to examine mRNA expression levels in HEK293 cells [26], however, most studies that have used microarray analyses on these cell lines have examined specific gene products of interest and reported data only for those gene products [20,26-28]. Their full data sets are available through NCBI, but are difficult for the non-specialist user to access and collate.

As a consequence, we sought to identify and make accessible the full complement of endogenously expressed non-chemosensory GPCRs of four cell lines used in GPCR research: the HEK293, AtT20, BV2 and N18 lines. Using microarray analysis we measured the mRNA levels from each of these four cell lines specifically identifying the expression levels of GPCRs and over a hundred of GPCR-related gene products and report them here.

## Results

**Figure 1:**
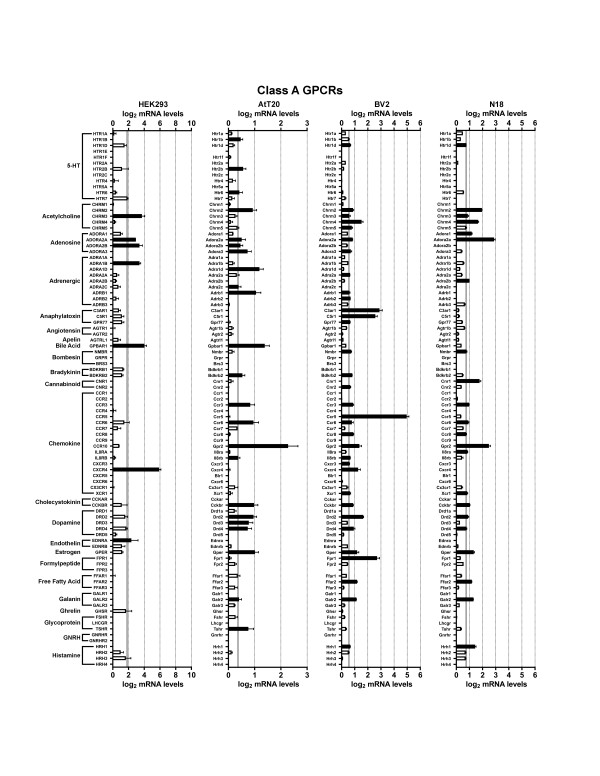
**mRNA expression levels of Class A GPCRs**. Microarray analysis of mRNA levels of Class A GPCRs with known ligands for HEK293, AtT20, BV2 and N18 cell lines. The gray line indicates the threshold of statistical significance and the dark bars are proteins for which statistically significant levels of mRNA were detected. White bars indicate levels of mRNA for proteins that were present, but did not reach statistical significance. Abbreviation: 5-HT = 5-hydroxytryptamine; GNRH = Gonadotrophin-releasing hormone.

**Figure 2:**
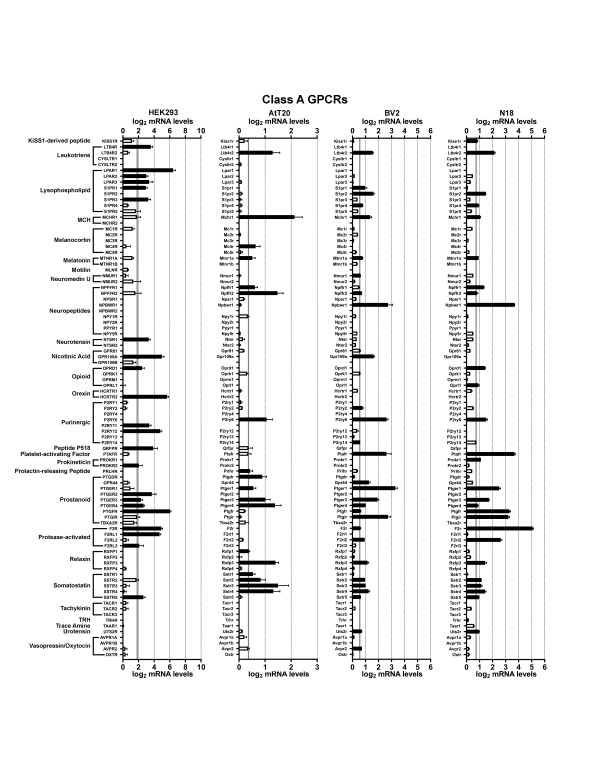
**mRNA expression levels of Class A GPCRs**. Microarray analysis of mRNA levels of Class A GPCRs with known ligands for HEK293, AtT20, BV2 and N18 cell lines. The gray line indicates the threshold of statistical significance and the dark bars are proteins for which statistically significant levels of mRNA were detected. White bars indicate levels of mRNA for proteins that were present, but did not reach statistical significance. Abbreviation: MCH = Melanin-concentrating hormone; TRH = Thyrotropin-releasing hormone.

**Figure 3:**
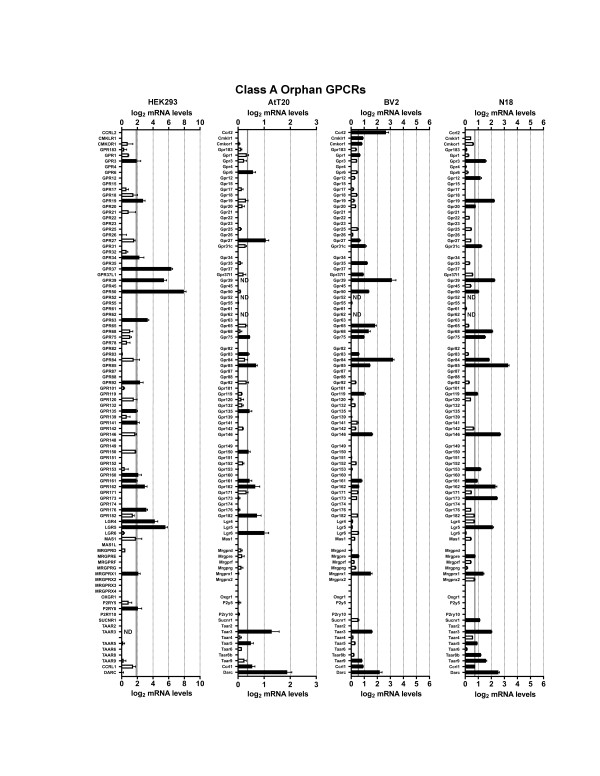
**mRNA expression levels of Class A Orphan GPCRs**. Microarray analysis of mRNA levels of Class A GPCRs with unknown ligands for HEK293, AtT20, BV2 and N18 cell lines. The gray line indicates the threshold of statistical significance and the dark bars are proteins for which statistically significant levels of mRNA were detected. White bars indicate levels of mRNA for proteins that were present, but did not reach statistical significance. Abbreviation: ND = not determined.

**Figure 4:**
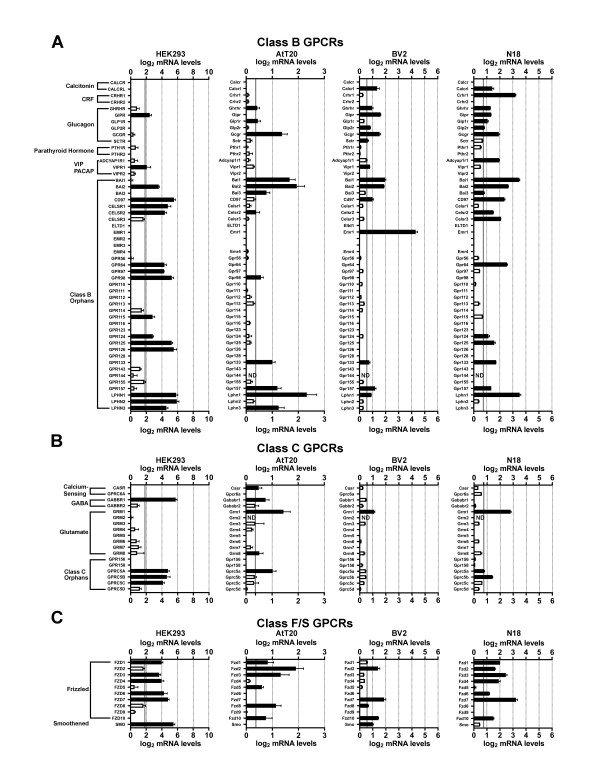
**mRNA expression levels of Class B, C, and F/S GPCRs**. Microarray analysis of mRNA levels of GPCRs with known ligands for HEK293, AtT20, BV2 and N18 cell lines. A: Class B GPCRs. B: Class C GPCRs. C: Class F/S GPCRs. The gray lines indicate the threshold of statistical significance and the dark bars are proteins for which statistically significant levels of mRNA were detected. White bars indicate levels of mRNA for proteins that were present, but did not reach statistical significance. Abbreviations: BAI = Brain-specific angiogenesis inhibitor; CELSR = Cadherin EGF LAG seven-pass G-type receptor; CRF = Corticotropin-releasing factor; ND = Not determined; PACAP = pituitary adenylyl cyclase-activating polypeptide; VIP = Vasoactive intestinal peptide.

**Figure 5:**
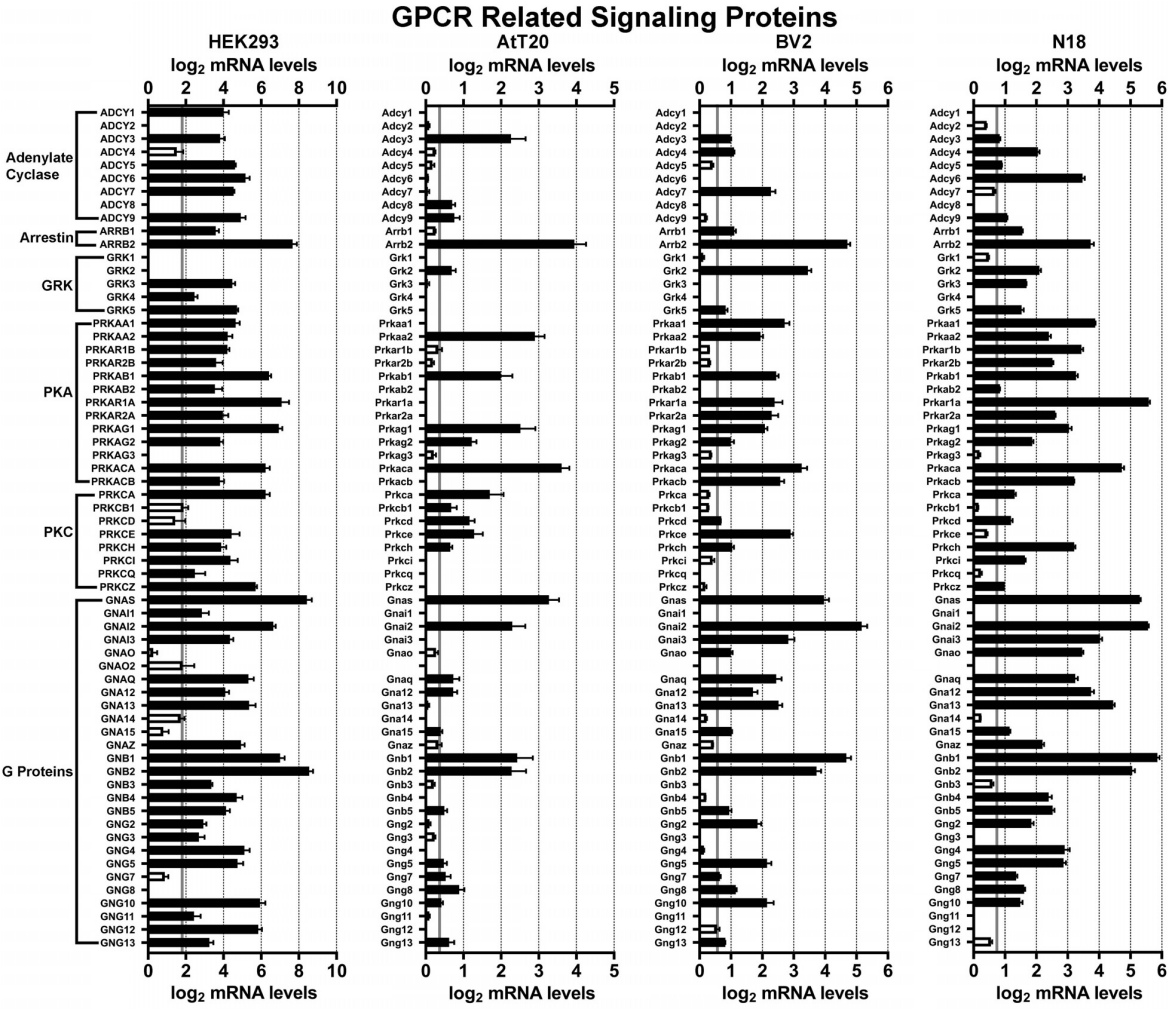
**mRNA expression analysis of GPCR related signaling proteins**. Microarray analysis of mRNA levels of GPCR related signaling proteins for HEK293, AtT20, BV2 and N18 cell lines. The gray lines indicate the threshold of statistical significance and the dark bars are proteins for which statistically significant levels of mRNA were detected. White bars indicate levels of mRNA for proteins that were present, but did not reach statistical significance. Abbreviations: GRK = GPCR Kinase; PKA = Protein kinase A; PKC = Protein kinase C.

**Figure 6:**
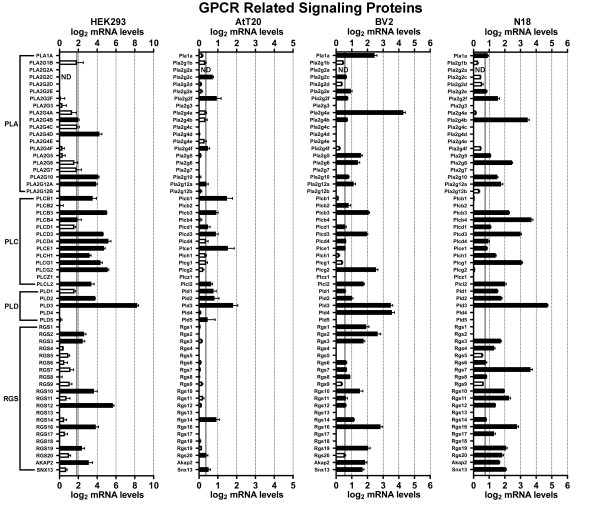
**mRNA expression analysis of GPCR related signaling proteins**. Microarray analysis of mRNA levels of GPCR related signaling proteins for HEK293, AtT20, BV2 and N18 cell lines. The gray lines indicate the threshold of statistical significance and the dark bars are proteins for which statistically significant levels of mRNA were detected. White bars indicate levels of mRNA for proteins that were present, but did not reach statistical significance. Abbreviations: ND = Not determined; PLA = Phospolipase A; PLC = Phospholipase C; PLD = Phospholipase D; RGS = Regulator of G protein signaling.

We first examined HEK293 cells, a cell line that was made by transforming human embryonic kidney cells with adenovirus type 5 DNA. Developed in 1977, HEK293 cells may be the single most widely used cell line for heterologous expression (both transient and stable expression) of GPCRs and other proteins of interest [17]. Figures 1,2,3,4,5 and 6 show the mRNA levels of GPCR and GPCR-related gene products found in HEK293 cells. Of Class A GPCRs, shown in figures 1 and 2, HEK293 cells express mRNA for several different types of receptors within specific families of receptors: multiple types of adenosine, lysophospholipid, purinergic, prostanoid, and protease-activated receptors, among others. In results relevant to the study of orphan GPCRs (figure 3), HEK293 cells contain high message levels of GPR37, 2 types of orphan-LGR (glycoprotein hormone) receptors--LGR4 and LGR5, GPR39--and very high levels of the melatonin-related receptor GPR50. HEK293 cells may therefore be suitable tools for deorphanization of these receptors. Interestingly, HEK293 cells contain relatively little mRNA for Class B GPCRs with known ligands (figure 4A), but quite high levels of many orphan Class B GPCRs. The same is true for Class C GPCRs as seen in figure 4B though they do appear to contain high levels of GABA_B_R1 receptor message, though lacking mRNA for GABA_B_R2, necessary to form a fully functional GABA-B receptor. Finally, for GPCRs, HEK293 cells contain a number of types of frizzled receptors as well as smoothened receptor (figure 4C). Figures 5 and 6 show the mRNA levels of various types of GPCR signaling-related gene products such as the G proteins and a number of their targets and downstream effectors as well as proteins that regulate GPCR signaling, the GRK, beta-arrestin (ARRB) and RGS proteins. Interestingly HEK293 cells express mRNA for numerous isoforms of adenylyl cyclase and protein kinases A and C (PRKA, PRKC) and phospholipase C (PLC), while having a limited diversity of phospholipase A (PLA) and RGS proteins. HEK293 cells also appear to have an almost full complement of G protein isoforms for each subunit, making these cells an attractive environment to express any number of GPCRs to study downstream G protein-mediated signaling.

Next we analyzed the AtT20 cell line, a neuroendocrine line derived from a mouse pituitary tumor that has neuron-like properties [29]. This cell line is often used as a model neuroendocrine cell line and has been a useful cell line to study of ion channel modulation by GPCRs [18,30,31]. Unlike HEK293 cells, there has been relatively little work performed to identify the GPCRs and related signaling molecules present in AtT20 cells. Our current results highlight that AtT20 cells express a diverse range of GPCR mRNAs. Figures 1 and 2 show that they possess a number of types of Class A receptors with a few isoforms of each type. According to our analysis here, they express a few different isoforms of adenosine, chemokine, dopamine, neuropeptide FF, prostanoid and somatostatin receptors with a number of other individual members from other types. Figure 3 shows that AtT20 cells have a limited breadth of Class A orphan GPCRs. In Class B, AtT20 cells express mRNA for a number of glucagon, orphan brain-specific angiogenesis inhibitor (BAI), and orphan latrophilin receptors (figure 4A). Like HEK293 cells, AtT20 cells express mRNA for GABA_B_R1 (with a detectable, but not significant level of GABA_B_R2 mRNA), but unlike HEK293s they express several metabotropic glutamate receptor genes (figure 4B). For Class F/S in AtT20s we detected mRNA for six frizzled receptors but not smoothened receptor mRNA (figure 4C). AtT20's have a somewhat sparser repertoire of GPCR signaling-related gene products than HEK293s, especially within the G protein subunits (figures 5 and 6).

We next examined BV2 cells, a cell line that is often used as a model system for the study of microglial function [32]. As shown in figures 1 and 2, BV2 cells predominately possess mRNA for anaphylatoxin, chemokine, and prostanoid Class A GPCRs as would be expected from an immune system-derived cell line. They also express mRNA for a scattering of other receptor types, such as a few muscarinic, somatostatin, and lipid receptors, among others. As has been reported in the literature, BV2 cells express CB2 cannabinoid receptors (Cnr2) [33], which makes them unique among the other cell lines examined here. We also observed the expression of a large number of different Class A orphan GPCR mRNAs as seen in figure 3, though a much less diverse repertoire of orphans from Class B (figure 4A) or Class C (figure 4B) receptors were present. BV2 cells express only one Class C GPCR at a significant, albeit, low mRNA level: the metabotropic glutamate receptor, mGluR1 (Grm1). Figure 4C shows that BV2 cells possess mRNA for a few different types of frizzled receptors as well as the smoothened receptor. BV2 cells have a wide complement of GPCR signaling-related gene products (figures 5 and 6). Though not quite as extensive as HEK293 cells, BV2 cells possess numerous isoforms of different phospholipases, PKA, PKC, and adenylyl cyclase. Their beta-arrestin, RGS protein, and GRK levels suggest the capacity to highly regulate GPCR signaling as well.

Lastly we investigated the N18 cell line. This cell line is derived from a neuroblastoma and is commonly employed as a neuron-like cell line. It has been used as a tool to analyze ion channel function in response to toxins and other regulators [19,34-37]. We found that N18 cells have mRNA for a broad range of different Class A GPCRs as seen in figures 1 and 2. N18 cells have mRNA for a few types of muscarinic acetylcholine, adenosine, chemokine, neuropeptides, prostanoids, protease-activated, and somatostatin receptors with a few other individual types such as CB_1 _cannabinoid receptors (Cnr1). Like the BV2 cells, N18 cells have mRNA for quite a few Class A orphan GPCRs (figure 3) with a similar profile as well. They further express mRNA for a broad complement of Class B GPCRs (figure 4A) but express a limited profile of Class C GPCRs (figure 4B). They also have mRNA for a number of different types of frizzled receptors, but lack mRNA for a smoothened receptor (figure 4C). N18 cells express high levels of many of the GPCR signaling-related gene products. They have abundant levels of G proteins and numerous isoforms of PKA. N18 cells' GPCRs are also presumably tightly regulated based on the assortment of GRK, RGS and beta-arrestin mRNAs detected (figures 5 and 6).

**Figure 7:**
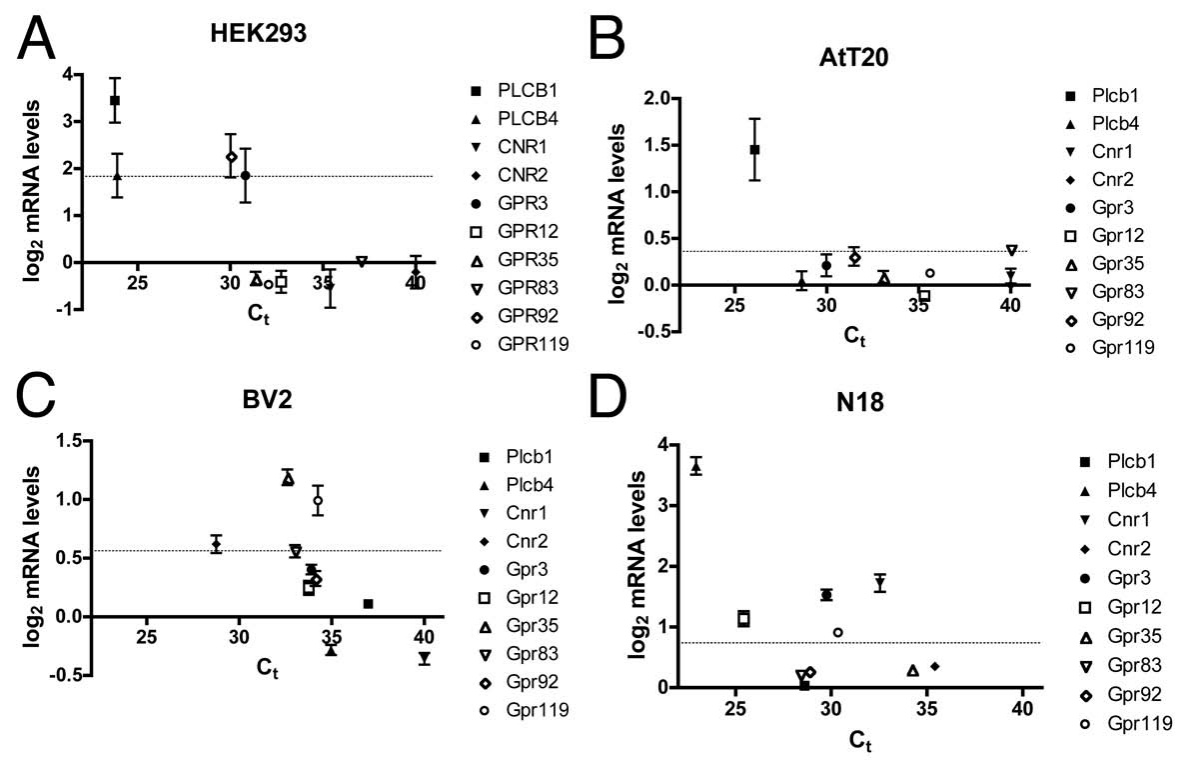
**Quantitative polymerase chain reaction validation of microarray analysis**. Quantitative PCR analysis of 10 genes from (A) HEK293, (B) AtT20, (C) BV2, and (D) N18 cell lines. mRNA levels determined from qPCR analysis were plotted against the mRNA levels measured in the microarray analyses in figures 1, 3 and 6. The dashed lines indicate the threshold of statistical significance for the microarray data. Points that fall between 0 and this threshold indicate genes that were detected with the microarray analysis but failed to meet statistical significance. Lower critical threshold (Ct) values indicate high mRNA copy numbers.

In order to validate our findings with the microarray analyses, we performed quantitative real-time PCR (qPCR) analysis on these four cell lines. We chose a range of GPCRs, mostly orphan receptors (Cnr1, Cnr2, Gpr3, Gpr12, Gpr35, Gpr83, and Gpr119), as well as two phospholipases (Plcb1 and Plcb4) as candidates to measure their mRNA levels using this alternative method. We selected genes that had distinct and differential expression profiles across the four cell lines. The data are plotted in figure 7 in a manner that correlates the copy number of mRNAs measured in each cell line using qPCR with the levels of mRNA determined from our microarray experiments. The qPCR data were broadly consistent with our microarray data. We observed higher copy numbers for all genes for which we had observed statistically significant microarray expression, suggesting an overall good correlation between our microarray and qPCR data. The qPCR did detect mRNA for a few genes whose levels of expression did not reach statistical significance in the microarray analyses. These genes are Plcb4, Gpr3, and Gpr92 in AtT20 cells and Plcb1, Gpr83 and Gpr92 in N18 cells.

## Discussion

The ability to induce expression of a known protein in a model cellular system, such as the cell lines analyzed here, has served as a remarkable, vital tool. However, the GPCR research community that makes use of these cell lines has had only limited data on the complement of GPCRs and related signaling machinery expressed in them. When a cell line is used for heterologous expression, ignorance of the endogenous protein expression profile of a cell line may lead to false conclusions about the nature of receptor function. Indeed, the cellular milieu can have a dramatic impact on GPCR signaling (see [22]. Microarray technology is now sufficiently accessible and mature to determine the mRNA expression for cell lines from species whose genomes have been sequenced. Yet even where microarray studies have been conducted, their data remains only nominally accessible to those with sufficient means, experience and time to extract useful information. The absence of a systematic published study of mRNA expression for signaling proteins of general interest hinders research using these important cell lines. This study provides data examining the presence of several hundred different GPCRs and related signaling proteins in four phenotypically diverse cell lines frequently used in GPCR research.

HEK293 cells are perhaps the most widely used cell line, cited in over four thousand publications and initial work has identified a number of endogenous receptors (see below). We expanded upon this knowledge by demonstrating that HEK293 cells express at least 75 different GPCR mRNAs. Much of what has been reported about GPCR mRNA expression patterns in HEK293 cells was confirmed by our analysis: ADORA2B, CHRM3, F2R, F2RL1, GABABR1, GPCR5A, GPR19, LGR5, LPAR1, LPAR2, P2YR1, PTGER2, S1PR, and SSTR5 [17,26,38-46]. HEK293 cells have been reported to possess mRNA for a number of receptors that were not found to be expressed at statistically significant levels in our study: 5HT1D, 5HT6, 5HT7, ADRA2(a-c), ADRAB2, AGTR1, BDKRB2, DRD2, GPR1, GPR161, GRM4, P2YR2 and SSTR2 [17,26,46-50]. Our results also indicate that HEK293 cells express mRNA for most G protein subunit isoforms, and numerous isoforms of adenylyl cyclase, protein kinases A and C, and phospholipase C. In addition they are useful for studying regulation of G protein mediated signaling as significant levels of mRNA for beta-arrestin1 and 2, GRK3-5 and a number of RGS subtypes were seen.

Much less is known of the expression patterns of GPCRs in AtT20 cells. We confirmed the presence of Sstr1 and Sstr2 mRNA in AtT20 cells [30], but did not observe significant levels of any of the other receptors that have previously been reported such as Chrm4 [51], Vipr1a, Adcyap1r1, or Sstr5 [30,52,53]. The lack of significant levels of CRF receptor mRNA was of interest as AtT20 cells have been reported to be sensitive to CRF [54]. mRNA for Hrh3 or Hrh4 histamine receptors were not detectable, despite reports of their presence [55,56], but a low level of Hrh2 was found. Tacr1 [31] was not detected, nor were Tacr2 and Tacr3. We also did not detect the orphan Cmkor1/Cxcr7 [57]. It is worth noting that there are at least two AtT20 clones available and this may explain some of the differences we found. Generally speaking, AtT20 cells possess a much less diverse complement of GPCR-related signaling and regulatory gene products than the other cell lines tested. Of the approximately 120 GPCR signaling gene products that we studied here, AtT20 cells had 45 significantly expressed signaling related proteins compared to 72 or more in each of the 3 other cell lines. AtT20 cells were the only ones to not have significant levels of beta-arrestin1 (Arrb1) or GRK3-5. Only three RGS gene products were found at significantly high levels: Rgs14, Rgs20, and Snx13.

BV2 cells are commonly used to study microglial biology. These cells exist in a continuum between two states: a resting state and an activated state. For example, upon interferon gamma treatment (primed state), BV2 cells increase both Cnr2 and Gpr55 mRNA levels, whereas with lipopolysaccharide treatment (activated state) these levels decrease [58]. In our case, BV2 cells were cultured under conditions expected to promote a resting state. We can confirm that as reported in the literature BV2 cells express Adora3 [59], Cnr2 [33,58], Mtnr1a [60], Galr2 [61], Ccr5, Cxcr3 [62] and Rgs2, 10, 12 [27,28,63]. We also detected mRNA for receptors that have been reported to be upregulated in response to stimulation such as Ccrl2 [28,64], Ptafr, Ptigr [64] and Rgs7 [65]. Here, too, we identified a few receptors that have been reported present for which we found levels of mRNA below the levels that we defined as statistically significant: Hcrtr1 [66], Gpr55 [58] and the receptor for Cx3cr1 [67]. We did not detect Prokr1 which is upregulated with activation [27], nor for Oprm1, P2ry1 and Gpr149 which are downregulated following activation [27,28]. Several reports have identified the presence of mGluRs in BV2 and primary microglia [21,68,69]. Interestingly, Grm1 is generally thought *not *to be present in BV2 cells, yet it was the only one that we significantly detected. We did find low, non-significant levels of Grm3, 6, and 8. The differences may be due to differences in the activation state of the cell lines. BV2 cells appear have a diverse receptor and signaling gene product profile.

Of the cell lines tested, the least well characterized is the N18 neuroblastoma line. They are known to express Cnr1 [70], which we confirmed with our analysis. It has been reported by some that they do not express muscarinic receptors [71], but our analysis shows that they have mRNA for three different types (Chrm2, 3, and 4) consistent with other reports [72,73]. These studies also found evidence for secretin, prostacyclin, opioid and alpha_2_-adrenergic receptors In support of this we found significantly high levels of mRNA for Ptgir1, Ptger1, Ptger3, Ptger4, Oprd1, Oprl1 and Adra2b. There were also levels of mRNA for Sctr, Adra2a, Oprk1 and Oprm1 that did not reach statistical significance. Another report describes P2ry2 expression [74], but for this we only found low non-significant levels. Beyond these reports there seems to be little known of GPCR signaling in this cell line and this analysis substantially extends our knowledge of its complement of GPCR expression, at least at the mRNA level. Like BV2 cells, N18 cells appear to have a large repertoire of GPCRs, effectors, and regulating proteins, being consistently one of the highest expressers in each receptor class analyzed.

Interestingly, even the most prolific cell line expressed only a small portion of the full repertoire of non-chemosensory GPCRs (~360). Of these four cell types, BV2 cells expressed the highest total number of GPCRs (108) followed by N18 (105), AtT20 (79), and HEK293 cells (73). BV2 cells had the greatest total abundance of Class A receptors (88), but had equal numbers of Class A orphan GPCRs as N18 cells (figure 3). On the other hand, HEK293 cells have mRNA for the greater number of Class B and Class C orphans (figure 4). It is interesting that the BV2 and the N18 cell lines, which are more often used as models for specific cell types, express mRNA for the most diverse set of receptors. This presumably results in a greater sensitivity to a broad spectrum of ligands and the large number of signaling proteins for which we measured high mRNA levels in these two cell lines attest to this. It is also of interest that HEK293 and AtT20 cells have the lowest numbers of GPCRs of these four lines. Low expression (both of amount and type) may allow these cell lines to serve as a better-differentiated cell model, and, from an experimental perspective, may make a given cell line more attractive as a 'clean slate' into which gene products of interest can be introduced.

mRNA expression levels do not guarantee that a specific mRNA is properly translated, folded, and trafficked to produce functional protein. Discrepancies between our findings and those that have been reported using PCR-based methods may be due to differences that appear in cell lines with time, passage number and growth conditions. Discrepancies may also be attributed to the PCR-based techniques generally employed to extract and amplify mRNA: in some cases the techniques used in many of the studies cited here, some of which do not allow for relative quantification, may have detected very low levels of receptor mRNA that did not reach our threshold for statistical significance. In light of the potential discrepancies between microarray and PCR-based techniques, we employed qPCR analysis to test the validity of our microarray results (figure 7). The overall consistency between our qPCR results and microarray data lend confidence to the concept that the microarray analyses accurately reflect the presence of mRNA for these GPCRs and their signaling proteins.

## Conclusions

In conclusion, this microarray analysis of the nearly full complement of non-chemosensory GPCRs and related signaling proteins in these four cell lines greatly expands our understanding of their potential signaling repertoire. These cell lines are used frequently as models of specific cell types, in heterologous expression, and as controls for antibody screening. The present work provides accessible profiles of mRNA expression that we hope will assist investigators of the GPCR superfamily in choosing the appropriate cell line to study a particular GPCR or related protein of interest and further to better understand potential functional interactions between GPCRs and related proteins in these lines. It is our hope that this work can eventually be extended to other commonly used cell lines such as CHO and COS7 cells when their host genomes (in this case hamster and green monkey, respectively) have been sequenced and made available for gene microarray analysis.

## Methods

### Cell cultures

Human Embryonic Kidney (HEK) (catalog #CRL-1573) and AtT20 (catalog #CRL-1795) cells were purchased from American Type Culture Collection (Boston, MA). BV2 microglia cells were obtained from Dr. Nephi Stella (University of Washington) [32,33]. N18 neuroblastoma cells were obtained from Dr. William Catterall (University of Washington)[75]. Dulbecco's modified Eagle's medium (DMEM), penicillin, streptomycin, and fetal bovine serum (FBS) were purchased from Gibco Life Technologies (Rockville, MD). All cell lines were grown in DMEM with 10% FBS, 100 units ml-1 penicillin, and 100 μg ml-1 streptomycin at 37°C in 5% CO_2 _humidified air. Importantly, BV2 cells were maintained in conditions that push BV2 cells towards a resting, non-activated state.

### Cell preparation, mRNA extraction & microarray analysis

Hybridization and sample imaging/quantification was performed by Indiana University's Center for Genomics and Bioinformatics. To study mouse and human mRNA expression, we used Nimblegen 4 × 72 k arrays (NCBI MM8, and NCBI HG18, respectively; 60 mer probe lengths). These are four-plex arrays that allow for the examination of mRNA expression of ~20,000 genes from four samples. The layout provides for three probes per target, the values of which we averaged to obtain a sample value. We cultured four 10 cm plates of each cell line, performing separate extractions when the cells grew to confluence. Total RNA was extracted from cells using TRIzol reagent (Invitrogen) under RNase-free conditions, followed by chloroform treatment (100 ul/ml). After centrifugation, the aqueous phase containing total RNA is then extracted and treated with isopropanol (500 uL/mL) for 5-10 mins. After centrifugation, the RNA pellet was isolated, washed with ethanol (75%; 1 ml/1 mL of reagent solution), followed by another centrifugation and air-dried. The RNA pellet was resuspended in RNase-free water. Total RNA quantity was assessed using NanoDrop ND-1000, and the Bioanalyzer 2100 determined the 28S/18S ratio for quality assessment. To synthesize targets for hybridization, we began with 10.0 μg of total RNA and used Invitrogen's SuperScript Double-Stranded cDNA Synthesis kit using oligo (dT) primer (Promega) followed by DNA labeling using 1 O.D. CY3-labeled random nonamer primer (TriLink Biotechnologies) and 100 U Klenow fragment 3' > 5' exo-(NEB) per 1.0 μg double-stranded cDNA. Single-color hybridization, post-hybridization washing and scanning were done with NimbleGen reagents and according to the NimbleGen's User's Guide for Expression Analysis. Images were acquired using a GenePix 4200A scanner with GenePix 6.0 software. The data from these arrays were extracted using the software NimbleScan 2.4 (Roche NimbleGen) and processed into PAIR files.

We used two separate NimbleGen high-density arrays for expression analysis (design name = HG18 60 mer expr 4plex and design name = MM8 60 mer expr X4). The 4-plex arrays each consisted of 4 × 72,000 isothermal long-oligo nucleotide probes that are 60 nucleotides in length. The first was based on HG18, Build 36 version from NCBI of *Homo sapiens *gene catalog, which represented 24,000 protein-encoding genes with 3 probes per target and 1 replicate, and the second was MM8 version from NCBI of *Mus musculus *gene catalog, which represented 18,869 protein-encoding genes with 3 probes per target and 1 replicate.

### Quantitative polymerase chain reaction analysis

Primers for selected GPCRs and enzymes were designed using Primer-Blast (http://www.ncbi.nlm.nih.gov/tools/primer-blast). The mouse gene was used as template in the primer design, and either mouse or human genome was used to screen for non-specific targets. Suitable primers were determined in this way and were commercially produced (Integrated DNA Technologies, Coralville, Iowa). AtT20, BV-2, HEK293 and N18 cell lines were grown on 10 cm dishes until each monolayer was 50-95% confluent. Total RNA was prepared and reverse transcribed using a Retroscript Kit as per manufacturer's protocol (Cat# AM1710; Ambion Inc. Austin, Texas). Two micrograms of total RNA from each line was reverse transcribed, samples were diluted and qPCR was performed using a Stratagene Mx3000P thermocycler and Sybr Green/Low Rox polymerase mix (Cat.# 95056-100, Quanta Biosciences, Gaithersburg, MD). In the case of N18, the "no RT" control showed significant genomic DNA contamination. To compensate for this, the total RNA for this sample was treated with DNase 1 for 1 hour and again purified just prior to RT. Primers for glutaraldehyde-3-phosphate dehydrogenase (GADPH) were used to make an internal control for each cell line with the threshold cycle set at 16. Once the standard critical threshold (Ct) was set, the relative expression levels for genes within each cell line were determined.

### Database information

All data is MIAME compliant and has been deposited in GEO (accession number GSE25901).

### Statistical analysis

For statistical analysis of the microarray data, we first transformed the values from the microarray into log2 format for ease of comparison and data representation [76]. We then obtained values for 10-11 assorted seminal proteins that were unlikely to be expressed in these cell lines. For the human-derived HEK293 cells these were: PYY2, PSP94, SPATA3, SPATA4, SPATA5, SPATA13, SPATS1, SPESP1, SPERT, ODF1, and SPAM1. For the three murine cell lines we selected: Svs2, Svs3, Svs5, Svs6, Svs7, Sva, Svp2, Sval1, Sval2, Sval3. We averaged the expression level of each of these proteins for each of the four plates to determine the mean and standard deviation of their expression. We took these mean values to represent zero expression. To determine statistically significant expression, we added the mean plus two standard deviations of the expression levels of these seminal proteins for each plate. We then subtracted this value from the value for each receptor and protein for which we obtained a signal. The mean plus two standard deviations was considered to be the baseline for our proteins of interest as any values at this level or higher would be considered statistically significant (95% confidence that the true mean of the seminal protein expression would fall within the mean plus two standard deviations). Thus any protein remaining with a positive expression value is considered to be significantly expressed [76]. All graphs and statistical analyses were generated using GraphPad Prism 4.0 software (Hearne Scientific Software, Chicago, IL).

## Abbreviations

CRF: corticotropin releasing factor; DMEM: Dulbecco's modified Eagle's medium; FBS: fetal bovine serum; GPCR: G Protein Coupled Receptor; GRK: G protein coupled receptor kinase; HEK: Human Embryonic Kidney; mGluR: metabotropic glutamate receptor; PCR: polymerase chain reaction; RGS: Regulator of G protein signaling.

## Authors' contributions

BKA carried out the drafting of the manuscript and the statistical analysis. JL performed the microarray analysis and assisted in drafting the manuscript. JWM performed the cell line preparation, mRNA extraction and qPCR experiments. KM participated in the design and coordination of the study. AS conceived of the study, participated in the design and coordination of the study, critical review of the manuscript, and performed the data extraction. All authors contributed to writing, reading, and approving the final manuscript.
